# Circulating Aggrecan, Biglycan, and Decorin as Biomarkers of Osteoarticular Alterations in Juvenile Idiopathic Arthritis—A Preliminary Study

**DOI:** 10.3390/ijms262412168

**Published:** 2025-12-18

**Authors:** Kornelia Kuźnik-Trocha, Katarzyna Winsz-Szczotka, Krystyna Olczyk, Anna Gruenpeter, Katarzyna Komosińska-Vassev

**Affiliations:** 1 Department of Clinical Chemistry and Laboratory Diagnostics, Faculty of Pharmaceutical Sciences in Sosnowiec, Medical University of Silesia, Jedności 8, 41-200 Sosnowiec, Poland; olczyk@sum.edu.pl (K.O.); kvassev@sum.edu.pl (K.K.-V.); 2 Department of Practical Laboratory Medicine, Faculty of Pharmaceutical Sciences in Sosnowiec, Medical University of Silesia, Jedności 8, 41-200 Sosnowiec, Poland; winsz@sum.edu.pl; 3 Department of Rheumatology, The John Paul II Pediatric Center in Sosnowiec, ul. G. Zapolskiej 3, 41-218 Sosnowiec, Poland; anna.gruenpeter@gmail.com

**Keywords:** aggrecan, biglycan, decorin, proteoglycans, biomarkers, juvenile idiopathic arthritis

## Abstract

Proteoglycans and their fragments have potential as diagnostic or theragnostic biomarkers to identify diseases characterized by dysregulated extracellular matrix remodeling, such as juvenile idiopathic arthritis (JIA). Therefore, our study aimed to evaluate the diagnostic utility of plasma proteoglycan profiles, namely, aggrecan, decorin, and biglycan, released from osteoarticular structures into the blood of children with juvenile idiopathic arthritis. These profiles are potential biomarkers of tissue destruction and/or indicators of the efficacy of therapy with the biologic agent etanercept (ETA). This study was conducted on 263 blood samples collected from 25 healthy children and 34 children at various stages of juvenile idiopathic arthritis disease: immediately after diagnosis, following treatment with disease-modifying antirheumatic drugs (DMARD) (methotrexate, sulfasalazine, and prednisone), and during 3, 6, 12, 18, and 24 months of therapy with etanercept. Quantitative levels of aggrecan, biglycan, and decorin were measured using ELISA kits. In children with JIA, plasma aggrecan levels were elevated at diagnosis, decreased after ineffective DMARD therapy, and increased again at 24 months of etanercept treatment despite clinical improvement. By contrast, biglycan levels were similar to those in healthy controls but decreased during etanercept therapy. Decorin levels were initially high in untreated and DMARD-treated patients but returned to normal after 24 months of biologic treatment. After considering these findings and the ROC analysis, we conclude that decorin appears to be a promising biomarker for diagnosing and monitoring etanercept therapy in JIA, and biglycan is a useful biochemical marker for assessing the effectiveness of ETA treatment.

## 1. Introduction

Proteoglycans (PGs) are ubiquitous biomolecules composed of glycosaminoglycan (GAG) chains covalently linked to core proteins. These complex protein–carbohydrate macromolecules are essential components of the extracellular matrix (ECM), where they regulate tissue mechanical properties, protect the ECM, sequester proteins, and modulate cell signaling [1,2]. Although PGs are found in intracellular, membrane-bound, and extracellular forms, the majority are extracellular [3,4].

Major extracellular proteoglycans include small leucine-rich proteoglycans (SLRPs), such as decorin (DCN) and biglycan (BGN), and members of the aggrecan family, including aggrecan (AGC) [2]. Aggrecan is the main proteoglycan in the ECM of articular cartilage. It binds non-covalently to hyaluronan (HA), forming large, hydrophilic macromolecular complexes that resist joint compression and confer viscoelastic properties to cartilage [5,6]. Decorin and biglycan are proteoglycans present in cartilage and bone that play key roles in ECM assembly and cell signaling [1,7,8]. SLRPs bind directly to multiple types of collagens and modulate fibrillogenesis; they interact with various cytokines, including transforming growth factor-beta (TGF-β) and tumor necrosis factor-alpha (TNF-α) [9,10]. DCN and BGN also regulate chondrocyte metabolism by controlling the availability of growth factors to cells [9]. Furthermore, all three cartilage proteoglycans help protect the ECM from proteolytic degradation [2].

Proteoglycans may be released from their supramolecular assemblies into a soluble form through both proteolytic and non-proteolytic mechanisms [11]. Proteolytic and prooxidative imbalances are among the recognized factors contributing to the degradation of extracellular matrix components in juvenile idiopathic arthritis (JIA) [12]. JIA is a chronic autoimmune disease that occurs in childhood, characterized by persistent arthritis and progressive damage to bone and joint structures [13]. The early onset and heterogeneity of JIA hinder timely diagnosis, and early therapeutic intervention is essential for minimizing or eliminating inflammation and preventing or delaying the progression of joint destruction.

Given the central role of PGs in ECM integrity, their degradation and subsequent release into the bloodstream may provide insight into ongoing osteoarticular damage. Therefore, this study aimed to evaluate the diagnostic utility of circulating levels of aggrecan, decorin, and biglycan as potential biomarkers of tissue destruction and/or indicators of therapeutic response in children with JIA treated with the biologic drug etanercept (ETA). The diagnostic and monitoring value of proteoglycans was assessed using ROC curve analyses. In addition, we measured levels of matrix metalloproteinase-12 (MMP-12) and advanced oxidation protein products (AOPPs), which are biochemical markers associated with PG degradation. Finally, we investigated the relationships between AGC, DCN, and BGN levels and the concentrations of MMP-12 and AOPP in the blood of children with JIA. This allowed us to evaluate the associations between PG levels and MMP-12/AOPP concentrations and better understand the mechanisms underlying tissue damage and oxidative stress in JIA.

## 2. Results

The studies conducted indicated that all assessed proteoglycans, i.e., aggrecan, decorin, and biglycan, undergo quantitative changes in the blood plasma of patients with juvenile idiopathic arthritis throughout the course of the disease or biological treatment, as presented in Table 1. At the same time, we identified no significant effect of sex on the plasma concentration of the assessed PG (*p* > 0.05) in any of the study groups, i.e., in healthy children or patients with JIA—including untreated children, those undergoing 3–6 months of disease-modifying therapy with antirheumatic drugs (DMARDs), and those treated with etanercept for 2 years—as summarized in Supplementary Table S1.

### 2.1. Assessment of Aggrecan Concentrations in the Blood of Healthy Children and Children with Juvenile Idiopathic Arthritis

**Figure 1:**
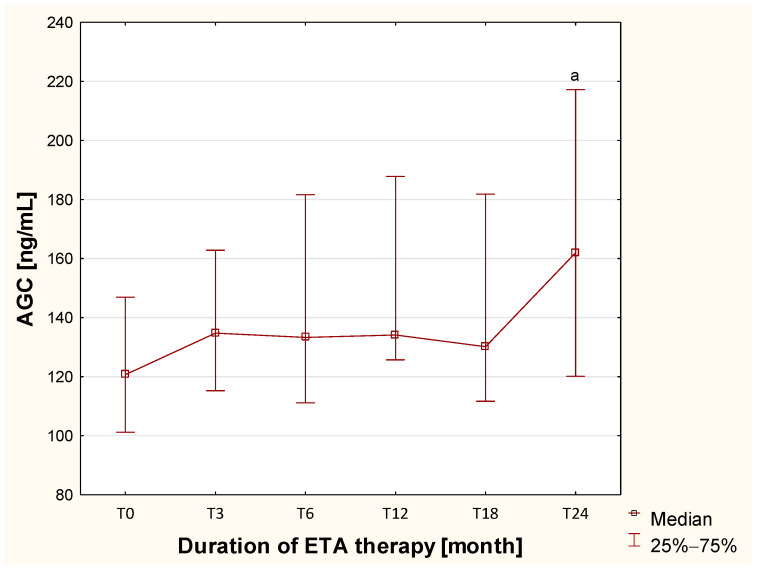
Changes in the concentration of aggrecan in the blood of children with JIA assessed before and throughout two years of ETA therapy. ^a^—Statistically confirmed difference versus the T0 group (*p* < 0.0033; Bonferroni-corrected).

As shown in Table 1, plasma aggrecan concentration increases significantly (*p* = 0.0029) in children with newly diagnosed or untreated JIA (TB) compared to healthy children (HC). Furthermore, treatment with DMARDs—methotrexate, sulfasalazine, and prednisone—which did not improve patients’ clinical condition (JADAS-27, Mdn = 35.5), led to a decrease in plasma aggrecan concentration (*p* = 0.0235). A different trend was observed during treatment with the biologic drug etanercept, as shown in Figure 1. We assessed the effect of etanercept treatment at the following time points: before biological therapy (T0) and after 3 (T3), 6 (T6), 12 (T12), 18 (T18), and 24 (T24) months of ETA ad-ministration.

The analysis showed that plasma AGC concentrations remained stable up to 18 months of biologic therapy, after which they increased sharply with statistical significance (*p* = 0.001), despite there being a marked clinical improvement in patients (JADAS-27, Mdn = 1.00).

**Figure 2:**
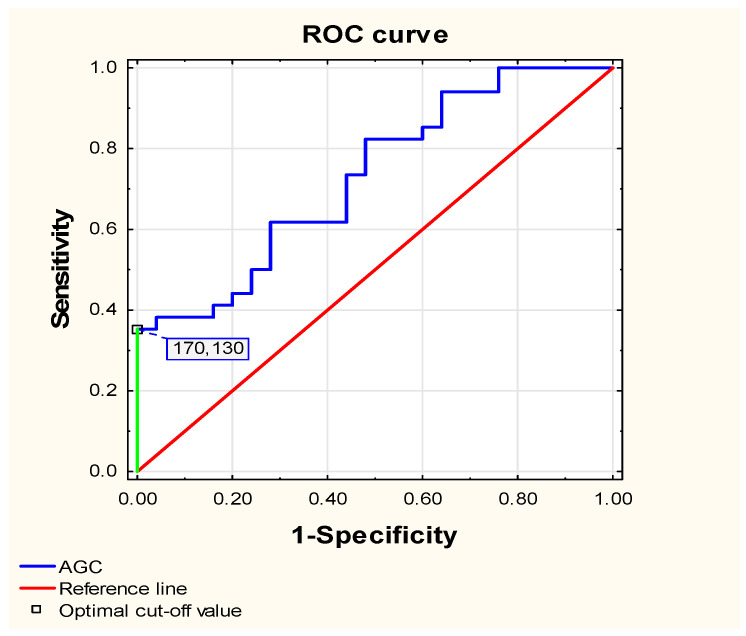
Receiver-operating characteristic (ROC) curve analysis for the diagnostic performance of serum aggrecan and its ability to distinguish patients with newly diagnosed, untreated JIA from healthy controls.

To assess the usefulness of AGC as a diagnostic marker for distinguishing between children with JIA and healthy children, logistic regression analysis was performed, adjusting for age, sex, and body mass index (BMI). AGC was identified as a significant predictor of disease status (B = 0.036, SE = 0.013, *p* = 0.005). Furthermore, ROC curve analysis (Figure 2) confirmed that AGC has a significant but moderate diagnostic ability in distinguishing between children with JIA and healthy children (AUC = 0.728, *p* = 0.0005).

At the optimal cutoff value of 170.130 [ng/mL], corresponding to a Youden index of 0.344, aggrecan showed high specificity (96.0%) but low sensitivity (38.2%), with an overall accuracy of 62.7%. The positive predictive value (92.9%) and negative predictive value (53.3%) indicate that AGC is specific and may serve as a confirmatory rather than a screening biomarker for JIA.

### 2.2. Assessment of Biglycan Concentrations in the Blood of Healthy Children and Children with Juvenile Idiopathic Arthritis

**Figure 3:**
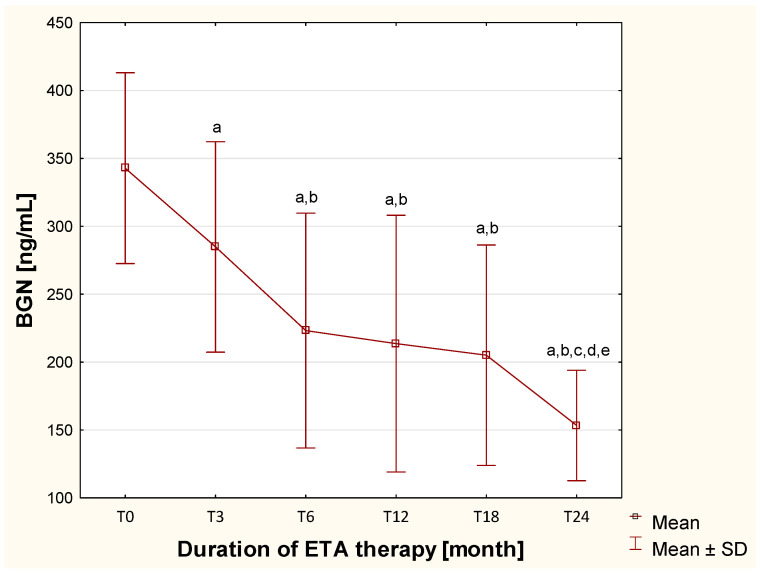
Changes in the concentration of biglycan in the blood of children with JIA, assessed before and throughout two years of ETA therapy. ^a^—statistically confirmed difference versus the T0 group (*p* < 0.0033; Bonferroni-corrected); ^b^—statistically confirmed difference versus the T3 group (*p* < 0.0033; Bonferroni-corrected); ^c^—statistically confirmed difference versus the T6 group (*p* < 0.0033; Bonferroni-corrected); ^d^—statistically confirmed difference versus the T12 (*p* < 0.0033; Bonferroni-corrected); ^e^—statistically confirmed difference versus the T18 group (*p* < 0.0033; Bonferroni-corrected).

Regarding the assessment of biglycan, plasma concentrations of this proteoglycan did not differ significantly (*p* > 0.05) between healthy children and those with JIA, including children both untreated (TB) and treated with DMARDs before biological therapy (T0) (Table 1). However, during biological treatment, plasma BGN concentrations significantly and systematically decreased (*p* < 0.0033) from the 6th to the 24th month of ETA therapy (Figure 3).

**Figure 4:**
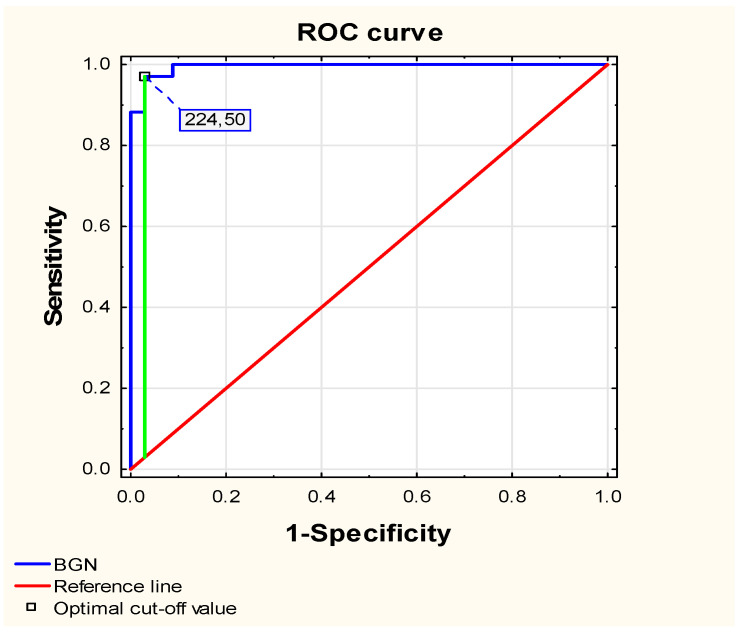
Receiver-operating characteristic (ROC) curves assessing the ability of changes in plasma bi-glycan level to distinguish between children with juvenile idiopathic arthritis before biological therapy (T0) and children after 24 months of etanercept treatment (T24).

We performed a multivariate regression analysis to assess the influence of age, sex, and BMI on changes in plasma biglycan concentrations in patients with JIA undergoing ETA treatment. The overall model was not statistically significant (F(3.30) = 0.21; *p* = 0.89); the predictors only accounted for 2.1% of the variance in BGN changes (R^2^ = 0.021). These results indicate that none of the studied variables had a statistically significant effect on changes in biglycan levels after treatment. Therefore, the observed decrease in BGN ap-pears to be independent of demographic and anthropometric factors and likely reflects the effects of biological therapy. The above results suggest the potential usefulness of measuring plasma biglycan concentrations in children with JIA as a biomarker for monitoring etanercept therapy. To confirm this, an ROC curve was constructed to assess whether changes in biglycan levels distinguish differences in patients before and after therapy (Figure 4).

ROC curve analysis showed that changes in biglycan concentration before (T0) and after etanercept treatment (T24) effectively distinguished between pre- and post-treatment states (AUC = 0.995, *p* < 0.00001), confirming the usefulness of biglycan as a marker for monitoring ETA therapy. The optimal cutoff value was 224.50 [ng/mL], corresponding to a Youden index of 0.94, and yielded a sensitivity of 97.1% and a specificity of 97.1%, resulting in an overall accuracy of 97.1%. In addition, both the positive and negative predictive values were 97.1%, confirming that BGN is a highly accurate biomarker with outstanding discriminative power.

### 2.3. Assessment of Decorin Concentrations in the Blood of Healthy Children and Children with Juvenile Idiopathic Arthritis

**Figure 5:**
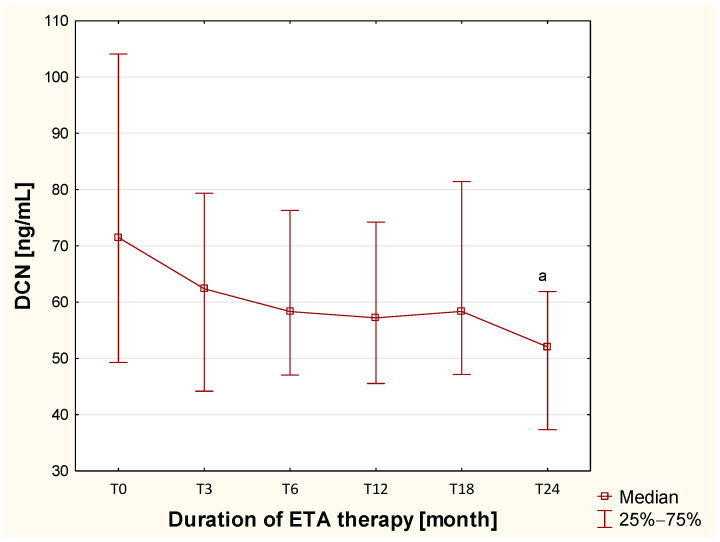
Changes in the concentration of decorin in the blood of children with JIA assessed before and throughout two years of ETA therapy. ^a^—statistically confirmed difference versus the T0 group (*p* < 0.0033; Bonferroni-corrected).

The authors found that decorin concentrations increased significantly in the blood of children with newly diagnosed, untreated JIA (*p* = 0.01) and in those treated with DMARDs before biological therapy (*p* = 0.0006), compared to healthy children (Table 1). Furthermore, no significant differences in plasma DCN concentrations were found between JIA patients in the TB and T0 groups (*p* > 0.05). However, plasma decorin concentrations gradually decreased with the duration of etanercept therapy, reaching near-normal levels after 24 months of ETA use (Figure 5).

**Figure 6:**
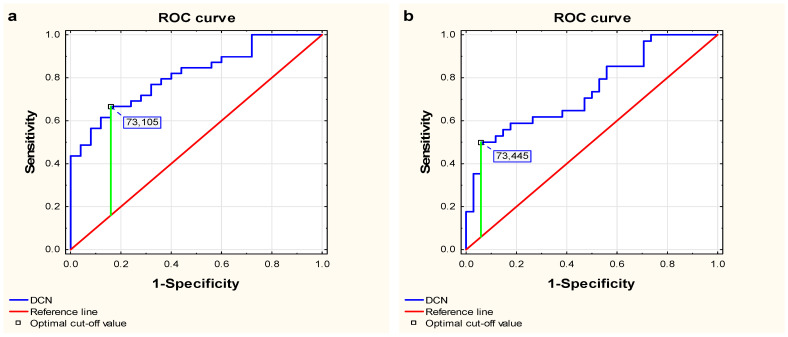
Receiver-operating characteristic (ROC) curves for decorin: (**a**) ROC curve illustrating the diagnostic performance of decorin in its ability to distinguish children with newly diagnosed, untreated JIA (TB) from healthy controls (HC). (**b**) ROC curve demonstrating the usefulness of decorin in monitoring therapy effectiveness based on measurements obtained before (T0) and after 24 months of etanercept therapy (T24).

These results suggest that, from the PGs studied, decorin may be a promising biochemical marker for diagnosis and monitoring the efficacy of etanercept therapy in juvenile idiopathic arthritis. To confirm the above hypothesis, ROC curve analyses were performed. The results showed that observed changes in decorin concentrations allowed for accurate differentiation between children with JIA and healthy children (AUC = 0.813, *p* = 0.026), as presented in Figure 6a, and moderate differentiation between patients before and after treatment (AUC = 0.743, *p* < 0.0001), as shown in Figure 6b.

For diagnostic purposes (Figure 6a), the optimal cutoff value of 73.105 [ng/mL] (Youden index = 0.51) yielded a sensitivity of 66.7%, a specificity of 84.0%, an overall accuracy of 73.4%, a positive predictive value of 86.7%, and a negative predictive value of 61.8%. This indicates that DCN may serve as a useful diagnostic biomarker.

In the context of monitoring treatment response (Figure 6b), at the optimal cutoff of 73.445 [ng/mL] (Youden index = 0.44), DCN had a sensitivity of 50.0% and a specificity of 94.1%, with an overall accuracy of 72.1%, a positive predictive value of 89.5%, and a negative predictive value of 65.3%. These findings suggest that DCN may serve as a complementary marker for monitoring therapeutic response, particularly due to its high specificity. However, its moderate sensitivity limits its use as a sole indicator of treatment efficacy.

In a logistic regression model, adjusting for age, sex, and BMI, decorin concentration was a significant predictor of disease (B = 0.063, SE = 0.019, *p* = 0.0011). Sex was another significant factor (B = 2.215, SE = 1.470, *p* = 0.038), with a higher probability of the disease occurring in girls after adjusting for decorin concentration. However, age (*p* = 0.764) and BMI (*p* = 0.344) did not show significant effects. Despite these results, the multivariate regression model did not identify a statistically significant effect for age, sex, or BMI on the change in decorin concentration (ΔDEC) during treatment with a biological drug (F(3.30) = 1.73, *p* = 0.182).

### 2.4. Assessment of Plasma Concentration on Proteolytic–Prooxidant Factors, i.e., MMP-12 and AOPP, and Their Relationship with Plasma Concentrations of Aggrecan, Biglycan, and Decorin

This study also assessed the plasma concentrations of selected proteolytic and prooxidant factors, i.e., matrix metalloproteinase-12 and biochemical markers of oxidative protein damage. As shown in Table 1, MMP-12 concentrations remained constant in the blood of all examined children, whether healthy or diagnosed with JIA (*p* > 0.05). However, AOPP concentrations changed throughout the course of JIA. For example, in newly diagnosed children with untreated JIA (TP), plasma AOPP concentrations increased (*p* = 0.00001) before decreasing (*p* = 0.0004) during DMARD therapy and remained stable throughout ETA treatment (*p* > 0.05). This differed from the values found in the blood of healthy children.

Assessing the relationships between the concentrations of determined proteoglycans (AGC, DCN, and BGN) and the measured markers of proteolytic–prooxidative imbalance (MMP-12, AOPP) in the blood of healthy children showed correlations between MMP-12 and AGC, between MMP-12 and DCN, and between AOPP and DCN (Table 2).

In turn, we analyzed correlations between the determined parameters in the blood of children with juvenile idiopathic arthritis, before treatment and after disease-modifying antirheumatic drug therapy (before biological therapy) and a 2-year course of etanercept. The results revealed only moderate negative correlations: between AGC and AOPP concentrations in children with newly diagnosed and untreated JIA, and between DCN and MMP-12 concentrations in children undergoing 24-month ETA therapy (Table 2).

### 2.5. Correlation Analyses

To further assess the biochemical changes that take place in juvenile idiopathic arthritis, we analyzed the relationships between plasma concentrations of aggrecan, bi-glycan, and decorin and inflammatory markers (erythrocyte sedimentation rate, ESR, and C-reactive protein, CRP), as well as the disease activity index (JADAS-27) in children with JIA. The results are presented in Table 3.

When assessing the relationship between disease activity and the concentrations of the studied proteoglycans, positive correlations were found between JADAS-27 index values and decorin concentrations in the blood of children with JIA in all studied groups, i.e., before treatment, after treatment with disease-modifying antirheumatic drugs and after 2 years of treatment with the biologic drug etanercept.

Further analysis of the relationship between nonspecific inflammatory markers and the concentrations of the studied proteoglycans demonstrated only a positive correlation between ESR values and aggrecan concentration in the blood of children with JIA treated for 24 months with ETA.

## 3. Discussion

Central to the pathogenesis of juvenile idiopathic arthritis is immune dysregulation, which leads to excessive production of proinflammatory cytokines (TNF-α, IL-1, IL-6, IL-17). These cytokines activate proteolytic enzymes such as matrix metalloproteinases and aggrecanases, which degrade key extracellular matrix components, including the proteoglycans aggrecan, decorin, and biglycan [14,15,16,17,18]. Oxidative stress, resulting from increased production of reactive oxygen species and impaired antioxidant defenses, further accelerates ECM breakdown [12,15,19]. Together, these sustained proinflammatory, proteolytic, and prooxidant processes contribute to the progressive, unrepaired destruction of joint cartilage in JIA [18].

While inflammation in affected children can be monitored with ultrasound and laboratory tests, detection of joint destruction requires clinical assessment combined with radiographic imaging. This method identifies changes in joint structure and joint space [20]. However, these techniques are relatively insensitive and primarily detect late-stage damage, which may negatively impact treatment outcomes and prognosis [20,21]. There-fore, new biomarkers are needed to improve the diagnosis and management of children with JIA.

Proteoglycans and their degradation products are promising biomarker candidates, as they reflect the balance between catabolic and anabolic processes within the extracellular matrix. These structural components, released into body fluids during progressive joint destruction, can serve as useful indicators of disease activity, as suggested by the proteoglycans analyzed in our study [20,21].

We observed significantly elevated plasma concentrations of aggrecan and decorin in children with JIA compared to healthy controls. Under physiological conditions, aggrecan is present in substantial amounts in almost all instances of articular cartilage—within pericellular (PCM), territorial, and interterritorial matrices, as well as chondrocytes—but is absent in fetal tissue [8]. Decorin, which is typically absent from the surface of joint cartilage, is present in deeper layers and bone tissue. DCN acts as a “physical linker”, strengthening the molecular association of aggrecan, and thereby increasing the structural integrity of the AGC network in the healthy cartilage extracellular matrix and reducing the loss of fragmented aggrecan from degenerative cartilage [8]. However, as our study indicates, in cases of significant tissue damage, such as that observed in children with newly diagnosed and untreated JIA, excess soluble aggrecan and decorin are released from the matrix into the blood. Although concentrations of aggrecan and decorin have not previously been assessed in the blood of children with juvenile idiopathic arthritis, the concentrations of their degradation products, i.e., aggrecan neoepitopes (ARGS) [20], dermatan sulfates (DS) [22], and keratan sulfates (KS) [23], have been studied. According to the literature, these aggrecan degradation products (KS and ARGS), as well as decorin (DS), are elevated in the blood of children with JIA [20,22,23]. These findings are, therefore, consistent with our study, which also demonstrated increased AGC and DCN concentrations in the blood of children with newly diagnosed JIA. Significantly higher AGC and DCN concentrations in the blood of untreated children with JIA suggest the potential of these PGs as diagnostic biomarkers. This hypothesis was partially confirmed by statistical analyses, which indicated that DCN can serve as the primary diagnostic marker (with significant diagnostic ability even after adjusting for sex), and AGC functions best as a complementary marker to confirm the diagnosis.

At the same time, our study did not reveal any changes in biglycan concentration in the blood of children with JIA, whether untreated or treated with DMARDs, compared to healthy children. This may be due to the location of PG in osteoarticular structures. Biglycan is found in deeper layers of cartilage (particularly the PCM) and in large amounts in bone tissue [8]. It is also known that biglycan plays a significant role in maintaining the structural integrity of subchondral bone [8]. Therefore, it is likely that the children studied had not yet developed sufficient bone tissue damage to increase this proteoglycan in the blood. Furthermore, in vitro studies on the progression of post-traumatic osteoarthritis indicate that decorin plays a more important role than biglycan in regulating cartilage degeneration, and biglycan is more important for regulating subchondral bone structure [24].

The decrease in aggrecan concentration observed in the blood of children with JIA after ineffective treatment with methotrexate, sulfasalazine, and prednisone in our study may be related to the effects of these drugs on proteoglycans. According to the literature, glucocorticoids tend to inhibit the expression, content, and/or structure of various PGs and their derived GAGs (particularly heparan sulfate [HS] and chondroitin sulfate [CS]) in most tissues and cells studied [1]. This may explain the different trends observed between aggrecan and decorin concentrations (the latter of which remains unchanged) in the blood of children with JIA after ineffective treatment with first-line drugs compared to untreated children. As mentioned above, the effects of glucocorticoids primarily involve inhibition of the expression of heparan sulfate proteoglycans (HSPGs) and chondroitin sulfate proteoglycans (CSPGs) [1]. Aggrecan is a representative CSPG that contains not only keratan sulfates but also significant amounts of chondroitin sulfates [9]. In contrast, decorin, a small leucine-rich proteoglycan, contains only a single GAG chain (either dermatan or chondroitin sulfate) [10].

The analysis of the usefulness of aggrecan, biglycan and decorin as markers of etanercept therapy efficacy confirmed the possibility of using only BGN and DCN as biomarkers. Concentrations of biglycan and decorin decreased progressively during biologic therapy. The mechanism likely responsible for the decrease in these PGs in blood concentrations is the inhibition of the inflammatory response mediated by TNF-α, and using its antagonist. This leads the metabolic balance to shift in favor of anabolic processes [25]. Reduced tissue degradation may manifest as a decrease in the release of both soluble BGN and DCN from the matrix into the blood. It is also important to note that the plasma concentration of decorin normalizes after two years of etanercept treatment compared with healthy children and participates in the reconstruction of damaged osteoarticular structures. However, when comparing the usefulness of decorin and biglycan as biomarkers for monitoring the effectiveness of etanercept treatment, BGN (AUC = 0.995) appears to be a significantly better biomarker than DCN (AUC = 0.743). Nonetheless, the positive correlations observed between plasma decorin concentrations and JADAS-27 scores in all studied groups of children with JIA indicate that decorin may serve as a marker of disease activity, significantly enhancing its potential.

Although changes in plasma aggrecan concentrations also occur in children with JIA receiving biological therapy, these are observed only after 2 years of treatment. The pattern observed in our cohort—elevated aggrecan levels in children with untreated JIA, stability during the first 18 months of etanercept treatment, followed by a sharp and significant increase after 24 months—is consistent with a two-phase model of cartilage response. During the first phase (0–18 months), biological therapy suppresses inflammation and halts ECM degradation but does not yet activate anabolic pathways. Only after prolonged clinical remission under TNF blockade—reflected by a marked improvement in JADAS-27 scores—do aggrecan concentrations increase sharply, exceeding levels observed in healthy children. This delayed overshoot is biologically plausible: once the inflammatory environment is stabilized, chondrocytes shift from a catabolic or survival mode to a reparative phenotype, initiating anabolic programs and producing proteoglycans at higher rates than those required for physiological homeostasis [26]. This enhanced synthesis reflects ECM remodeling during the restoration of cartilage integrity. This interpretation is consistent with recent evidence showing that chondrocytes retain the capacity to re-enter regenerative, anabolic states under sustained anti-inflammatory conditions [27]. Thus, the late increase in plasma aggrecan is best interpreted not as a pathological change but as evidence of a delayed, compensatory anabolic response during cartilage repair. Further studies are needed to validate this hypothesis.

As previously mentioned, the metabolism of cartilage proteoglycans is regulated by proteolytic enzymes, including ADAMTS-4/5 and various matrix metalloproteinases, as well as reactive oxygen species [28,29,30,31,32,33,34,35]. In JIA, excessive activity of these enzymes and ROS accelerates ECM degradation [12,18,28,29]. Among the MMPs involved, MMP-12 (macrophage elastase) is known to degrade aggrecan, biglycan, and decorin [28]; however, it remains a largely unexplored marker in JIA. Our studies did not detect changes in the concentration of this proteolytic enzyme in the blood of children with JIA. Although MMP-12 could degrade proteoglycans, its stable plasma levels in our study suggest that it may act locally within synovial tissue rather than systemically. Interestingly, we observed a weak negative correlation between plasma MMP-12 concentration and decorin levels in children with JIA who underwent 2 years of etanercept therapy. The same correlation was also found in healthy children, which may indicate normalization of DCN metabolic disorders under the influence of biological treatment in JIA patients.

Analysis of protein oxidation end products showed that their concentration in the blood of untreated children with JIA was increased compared to healthy controls. This was followed by a significant decrease in concentration after treatment with methotrexate, sulfasalazine, and prednisone. In contrast, plasma AOPP concentrations did not change after 24 months of etanercept therapy and remained only slightly higher than baseline, with no significant differences relative to healthy children. Because AOPP reflects the se-verity of oxidative stress [35], these findings suggest that a mild residual level of oxidative activity may persist in patients with JIA despite clinical improvement and inhibition of excessive cartilage degradation during treatment.

### Study Limitations

Some limitations of this study should be acknowledged, including the relatively small sample size, which limits the generalizability of our findings. Another limitation is the lack of assessment of pubertal status; however, given the age range of participants (4 to 13 years), they were likely prepubertal. Furthermore, the lack of a control group that improved clinically with DMARDs and the absence of a group treated with other biologics constitute additional limitations of this study. Importantly, the absence of comparative biologics restricts the generalizability of our findings to etanercept and precludes direct comparisons with other biologic therapies. In addition, cytokine profiling was not per-formed, which prevented the assessment of correlations between proteoglycans and established inflammatory biomarkers. Future studies should also include a longitudinal assessment of synovial fluid proteoglycans and their specific degradation products to better elucidate tissue–plasma relationships.

## 4. Materials and Methods

### 4.1. Samples

The study material consisted of 263 blood plasma samples that had been frozen and stored at −80 °C. The samples were collected for use in previous studies [15,23], including 25 healthy children and 34 children with juvenile idiopathic arthritis at the following stages of disease: immediately after diagnosis, after treatment with disease-modifying drugs (methotrexate, sulfasalazine, and prednisone), and during various months of therapy with a biological agent, such as a TNF-α inhibitor (etanercept).

### 4.2. Subjects

The observational cohort consisted of 34 children of both sexes, including 22 girls and 12 boys, aged 5 to 13 years old (pubertal children were excluded from the study), diagnosed with juvenile idiopathic arthritis according to the criteria of the International League of Associations for Rheumatology (ILAR) [36]. The diagnosis was made based on the duration of the disease (a minimum of 6 weeks), clinical symptoms (joint pain and swelling, limited joint mobility, and growth disturbances), and laboratory test results (CRP level, ESR value, presence of RF factor, and ANA antibodies). In children with JIA, disease activity was also assessed using the Juvenile Arthritis Disease Activity Score (JADAS-27), ranging from 0 to 57, which includes four variables: the physician’s global assessment of disease activity, the child’s or parent’s global assessment of the child’s well-being, the number of active joints (out of 27 assessed joints), and the ESR value [37].

**Figure 7:**
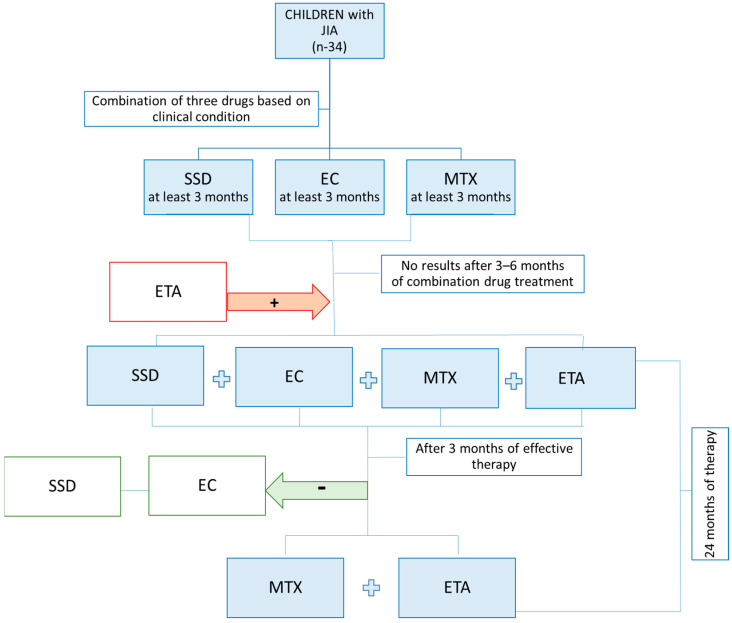
Treatment scheme for all patients with JIA (EC, prednisone; ETA, etanercept; MTX, methotrexate; SSD, sulfasalazine).

All JIA patients (n = 34) included in the study received the same treatment regimen, as illustrated in Figure 7 and described below.

Depending on the clinical condition and test results, children with JIA qualified for treatment with an appropriate disease-modifying antirheumatic drug (DMARD). Treatment included sulfasalazine (25 mg/kg/day), prednisone (up to 1 mg/kg/day with gradual tapering), and methotrexate (10–20 mg/m^2^ body surface area per week). Children with JIA in whom the above-mentioned treatment failed to improve their clinical condition, despite the use of a combination of two disease-modifying antirheumatic drugs/immunosuppressants at current doses (including methotrexate) for at least 3 months each, qualified for therapy with a biologic agent—the TNF inhibitor etanercept—as part of the Polish Therapeutic Program [38]. The program, designated as B.33, included patients over 4 years of age who met the following diagnostic criteria: (a) polyarticular JIA with at least 5 swollen joints and at least 3 joints with limited mobility and tenderness, accompanied by elevated ESR or CRP levels and disease activity assessed by a physician as ≥4 points on a 10-point scale, and (b) oligoarticular JIA, extended and persistent for more than 6 months, with poor prognostic factors (according to the American College of Rheumatology, ACR) and with at least 2 swollen joints or joints with limited mobility and tenderness, as well as disease activity assessed by a physician as ≥5 points on a 10-point scale [39]. Biological therapy consisted of subcutaneous injections of etanercept administered twice weekly at 3–4-day intervals at a dose of 0.4 mg/kg body weight (up to a maximum of 25 mg) or once weekly at 0.8 mg/kg body weight (up to a maximum of 50 mg). In all patients, etanercept was administered together with methotrexate, sulfasalazine, and prednisone at the start of treatment. After 3 months of effective therapy, sulfasalazine and prednisone were discontinued, while etanercept and methotrexate were continued for 24 months. The 2-year biologic therapy resulted in clinical improvement in all children, as assessed using the ACR Pediatric 30 criteria and/or the JADAS-27 score. Based on ACR Pediatric 30 criteria, clinical improvement in children treated with etanercept was observed when, after at least 11 months of biologic therapy (mean 11.26 ± 0.75 months from treatment initiation), resolution of active synovitis and extra-articular manifestations was confirmed, CRP and ESR levels returned to normal reference ranges, the physician’s global assessment of disease activity improved, and morning stiffness lasted less than 15 min. On the JADAS-27 scale, clinical improvement was defined as scores ≤ 1 for the oligoarticular subtype and ≤4 for the polyarticular subtype.

The control group consisted of 25 healthy children (14 girls and 11 boys), who were age-matched to the children with JIA, undergoing scheduled preventive examinations. The reference group included children whose routine laboratory test results (blood count, ESR, cholesterol, glucose, creatinine, and C-reactive protein) were within the normal reference ranges for their age group. Exclusion criteria included a history of illnesses requiring hospitalization within the previous year, surgical procedures, or chronic pharmacological treatment.

Body weight and height were measured, and BMI was calculated for all children. The demographic, anthropometric, and clinical characteristics of both healthy children and those with JIA—untreated, treated with DMARDs, and receiving biological therapy—are presented in Table 4.

The parents/legal guardians of both healthy children and those with JIA provided consent for the collection and use of any biological material (venous blood) remaining after the required diagnostic tests. The study was conducted in accordance with the Declaration of Helsinki. The protocol was approved by the Local Bioethics Committee of the Silesian Medical University in Katowice (BNW/NWN/0052/KB/168/I/18/23 approved on 13 April 2023).

### 4.3. Biochemical Studies

Quantitative determinations of aggrecan, biglycan, decorin, metalloproteinase 12, and biochemical markers of oxidative protein damage in the blood of children from the control and study groups were performed in three series, with each test conducted on the same day to minimize inter-study variability. In children with juvenile idiopathic arthritis, the concentrations of AGC, BGN, DCN, MMP-12, and AOPP were measured before drug administration (TB); after the administration of disease-modifying antirheumatic drugs but prior to biologic therapy (T0); and at subsequent time points during etanercept treatment: the third (T3), sixth (T6), twelfth (T12), eighteenth (T18), and twenty-fourth (T24) months of therapy.

Enzyme-linked immunosorbent assays (ELISAs) were used to quantify AGC, BGN, DCN, MMP-12, and AOPP according to the manufacturer’s protocol. All ELISA kits were intended for research use only and were obtained from Cloud-Clone Corp. (Katy, TX, USA). Plasma AGC concentration was assessed using the Human Aggrecan ELISA Kit (minimum detection limit: 0.55 ng/mL); BGN concentration was assessed using the Human Biglycan ELISA Kit (0.127 ng/mL); DCN was assessed using the Decorin ELISA Kit (0.44 ng/mL); MMP-12 was assessed using the Human Matrix Metalloproteinase 12 ELISA Kit (0.122 ng/mL); and AOPP was assessed using the Advanced Oxidation Protein Products ELISA Kit (23.3 ng/mL). Intra-assay variability for all parameters tested was less than 10%.

### 4.4. Statistical Analysis

Statistical analyses were performed using the Statistica 13.3 software package (TIBCO Software Inc., Kraków, Poland). The procedure involved several steps: checking the normality of each variable with the Shapiro–Wilk test, evaluating homogeneity of variances with the Snedecor–Fisher test, and computing descriptive statistics. Variables with a normal distribution were summarized as the mean ± standard deviation (SD), and variables that did not meet the normality assumption were reported as the median (Mdn) and interquartile range, defined by the first (Q1) and third quartiles (Q3). The significance of differences between the control and study groups was tested as follows: (a) for normally distributed variables, one-way ANOVA was used followed by Dunnett’s post hoc test; (b) for non-normally distributed variables, the nonparametric Kruskal–Wallis test was used followed by the Mann–Whitney U test for pairwise comparisons. The significance of differences between multiple time points within the study group (different months of treatment) was tested using repeated-measures ANOVA; for non-normally distributed variables, the Friedman test for dependent samples was used, followed by appropriate post hoc tests for multiple comparisons. An appropriate Bonferroni correction was applied for multiple comparisons in post hoc analyses. In addition, ROC curve and multiple regression analyses were performed to evaluate the parameters studied. The strength of correlation between two variables was assessed using Spearman’s rank correlation coefficient (R). A *p*-value < 0.05 was considered statistically significant.

## 5. Conclusions

From the proteoglycans analyzed, decorin showed the strongest combined diagnostic and monitoring potential. This is evidenced by its markedly elevated baseline levels, normalization during etanercept therapy, and robust diagnostic performance in ROC analysis. Biglycan, characterized by a systematic decrease in concentration during treatment and a clear ability to distinguish clinical improvement in ROC analysis, appears to be the most reliable indicator of therapeutic response. These results highlight decorin and biglycan as promising biomarkers in JIA patients treated with etanercept, although confirmation in larger cohorts is necessary.

## Figures and Tables

**Table 1:** Plasma concentrations of aggrecan, decorin, biglycan, matrix metalloproteinase 12, and advanced oxidation protein products in healthy children and those with JIA.

Parameter	Healthy Children	Children with JIA		
	HC (n = 25)	TB (n = 34)	T0 (n = 34)	T24 (n = 34)
AGC [ng/mL]	105.39 * (91.99; 120.34)	128.65 *^,a^ (108.49; 175.44)	120.68 *^,b^ (101.18; 146.95)	161.99 *^,a,c^ (120.13; 217.22)
BGN [ng/mL]	313.99 ± 87.42	294.82 ± 46.38	342.78 ± 70.22 ^b^	153.39 ± 40.66 ^a,b,c^
DCN [ng/mL]	54.20 * (37.58; 63.54)	81.77 *^,a^ (61.39; 100.69)	71.51*^,a^ (49.29; 104.11)	52.07 *^,b,c^ (37.35; 61.90)
AOPP [μg/mL]	5.87 * (5.01; 7.33)	8.94 *^,a^ (8.24; 10.60)	6.31 *^,b^ (5.63; 8.24)	7.05 *^,b^ (5.85; 8.60)
MMP-12 [ng/mL]	20.64 ± 4.75	20.26 ± 3.57	21.49 ± 4.92	20.76 ± 6.44

Results are expressed as mean ± SD; * results are presented in the form of a median and quartile range (Q1—first quartile; Q3—third quartile); ^a^—statistically significant difference compared to the control group (*p* < 0.0167; Bonferroni-corrected); ^b^—statistically significant difference compared to children with JIA before treatment (*p* < 0.05); ^c^—statistically significant difference compared to children with JIA before biological treatment (*p* < 0.05). HC, healthy children; TB, children with newly diagnosed or untreated JIA; T0, children with JIA treated with DMARDs before biological therapy; T24, children with JIA after 24 months of biological therapy.

**Table 2:** Spearman rank correlations between the concentrations of aggrecan, decorin, and biglycan and the concentrations of matrix metalloproteinase-12 and advanced oxidation protein products in the blood of healthy children and patients with JIA.

Parameter Tested		AOPP [μg/mL]				MMP-12 [ng/mL]			
		HC	TB	T0	T24	HC	TB	T0	T24
AGC [ng/mL]	R	NS	−0.399	NS	NS	0.410	NS	NS	NS
	*p*		0.020			0.042			
BGN [ng/mL]	R	NS	NS	NS	NS	NS	NS	NS	NS
	*p*								
DCN [ng/mL]	R	−0.492	NS	NS	NS	−0.473	NS	NS	−0.398
	*p*	0.012				0.017			0.020

HC, healthy children; TB, children with newly diagnosed and untreated JIA; T0, children with JIA treated with DMARDs before biological therapy; T24, children with JIA after 24 months of biological therapy; R, Spearman’s rank correlation coefficient; NS, not significant; *p*-value < 0.05.

**Table 3:** Spearman rank correlations between aggrecan, decorin, and biglycan levels and disease activity markers in children with JIA.

Parameter Tested		CRP [mg/L]			ESR [mm/h]			JADAS-27		
		TB	T0	T24	TB	T0	T24	TB	T0	T24
AGC [ng/mL]	R	NS	NS	NS	NS	NS	0.408	NS	NS	NS
	*p*						0.017			
BGN [ng/mL]	R	NS	NS	NS	NS	NS	NS	NS	NS	NS
	*p*									
DCN [ng/mL]	R	NS	NS	NS	NS	NS	NS	0.513	0.416	0.655
	*p*							0.002	0.014	0.000

TB, children with newly diagnosed and untreated JIA; T0, children with JIA treated with DMARDs before biological therapy; T24, children with JIA after 24 months of biological therapy; ESR, erythrocyte sedimentation rate; CRP, C-reactive protein; JADAS-27, disease activity index; R, Spearman’s rank correlation coefficient; NS, not significant. *p*-value < 0.05.

**Table 4:** Demographic and clinical characteristics of healthy children and children with JIA.

Parameter	Healthy Children	Children with JIA		
	HC (n = 25)	TB (n = 34)	T0 (n = 34)	T24 (n = 34)
Age [years]	8.76 ± 2.93	8.65 ± 2.65	8.65 ± 2.65	10.65 ± 2.65
Sex [girls/boys]	14/11	22/12	22/12	22/12
BMI [kg/m^2^]	20.94 ± 4.39	18.54 ± 4.86	18.13 ± 3.88	18.88 ± 3.88
WBC [10^3^/μL]	6.88 ± 2.20	8.21 ± 2.56	7.96 ± 2.31	7.57 ± 2.27
RBC [10^6^/μL)	4.64 ± 0.38	4.42 ± 0.41	4.39 ± 0.50	4.62 ± 0.46
Hb [g/dL]	13.15 ± 1.02	12.43 ± 1.53	12.25 ± 1.25	12.88 ± 1.36
Ht [%]	39.02 ± 3.02	37.93 ± 3.98	36.93 ± 3.63	37.82 ± 3.28
PLT [10^3^/μL]	322.33 ± 62.59	341.00 ± 91.90	326.24 ± 98.12	336.46 ± 101.26
TCH [mg/dL]	138.00 ± 15.37	129.31 ± 14.62	126.31 ± 13.38	148.22 ± 12.43
Glucose [mg/dL]	82.52 ± 8.28	90.64 ± 14.74	90.80 ± 20.77	89.24 ± 8.11
Cr [mg/dL]	0.44 ± 0.13	0.47 ± 0.15	0.44 ± 0.16	0.53 ± 0.14
CRP [mg/L]	1.46 (0.90–2.06) *	5.72 (1.84–22.80) *	3.02 (1.12–11.91) *	0.83 (0.46–6.08) *
ESR [mm/h]	5.00 (4.00–8.00) *	23.50 (13.00–34.00) *	20.50 (10.00–26.00) *	12.00 (8.00–18.00) *
ANA	-	62% (positive)	62% (positive)	62% (positive)
RF	-	100% (negative)	100% (negative)	100% (negative)
JIA subtype [oligoarticular/polyarticular]	-	15/19	15/19	15/19
Duration of the disease [month]	-	-	4.41 ± 1.52	28.41 ± 1.52
JADAS-27	-	36.50 (32.00–42.00) *	35.50 (29.00–38.00) *	1.00 (0.00–2.00) *
Drugs	-	-	MTX, EC, SSD	ETA, MTX

Results are expressed as mean ± SD; * results are presented in the form of a median and quartile range (Q_1_—first quartile; Q_3_—third quartile). HC, healthy children; TB, children with newly diagnosed, untreated JIA; T0, children with JIA treated with DMARDs before biological therapy; T24, children with JIA after 24 months of biological therapy; BMI, body mass index; WBC, white blood cell; RBC, red blood cell; Hb, hemoglobin; Ht, hematocrit; PLT, platelet; TCH, total cholesterol; Cr, creatinine; CRP, C-reactive protein; ESR, erythrocyte sedimentation rate; ANA, antinuclear antibodies; RF, rheumatoid factor; JADAS-27, disease activity index; MTX, methotrexate; SSD, sulfasalazine; EC, prednisone.

## Data Availability

The original data presented in the study are openly available in the Repository of the Medical University of Silesia in Katowice (Polish Medical Platform of the Medical University of Silesia) at DOI:10.71804/bfmk-fp76/ https://ppm.sum.edu.pl/info/researchdata/SUM812e328cfabf4dbfac904fa35d05759b/ (accessed on 5 November 2025).

## Supplementary Materials

The supporting information can be downloaded at https://www.mdpi.com/article/10.3390/ijms262412168/s1.

## Abbreviations

The following abbreviations are used in this manuscript:

ACR	American College of Radiology
ADAMTS	aggrecanases
AGC	aggrecan
ANA	antinuclear antibodies
AOPP	advanced oxidation protein products
ARGS	aggrecan neoepitopes
AUC	the area under the ROC curve
BGN	biglycan
BMI	body mass index
Cr	creatinine
CRP	C-reactive protein
CS	chondroitin sulfate
CSPG	chondroitin sulfate proteoglycan
DCN	decorin
DMARDs	disease-modifying antirheumatic drugs
DS	dermatan sulfate
EC	prednisone
ECM	extracellular matrix
ELISA	enzymatic immunoassays
ESR	erythrocyte sedimentation rate
ETA	etanercept
GAGs	glycosaminoglycans
HA	hyaluronic acid
Hb	hemoglobin
HC	healthy children
Ht	hematocrit
HS	heparan sulfate
HSPG	heparan sulfate proteoglycan
IL	interleukin
ILAR	International League of Associations for Rheumatology
JADAS-27	Juvenile Arthritis Disease Activity Score 27
JIA	juvenile idiopathic arthritis
KS	keratan sulfate
MMP	matrix metalloproteinase
MTX	methotrexate
PCM	pericellular matrix
PGs	proteoglycans
PLT	platelet
RBC	red blood cell
RF	rheumatoid factor
ROC	receiver-operating characteristic
ROS	reactive oxygen species
SLRPs	small leucine-rich proteoglycans
SSD	sulfasalazine
TCH	total cholesterol
TGF-β	transforming growth factor-beta
TNF-α	tumor necrosis factor-alpha
WBC	white blood cell
