# Supplementary material for: Circulating Aggrecan, Biglycan, and Decorin as Biomarkers of Osteoarticular Alterations in Juvenile Idiopathic Arthritis—A Preliminary Study
Source: Int J Mol Sci. 2025 Dec 18;26(24):12168. doi: 10.3390/ijms262412168 (PMC12733554; doi:10.3390/ijms262412168)
Supplement: Supplementary file 1 [file ijms-26-12168-s001.zip › ijms-4008237-supplementary.pdf]

Table S1. Plasma concentrations of aggrecan, decorin, biglycan, matrix metalloproteinase 12, and advanced oxidation protein products in healthy children and those with JIA, depending on sex.

**Study group**	**Sex**	**AGC** [ng/mL]	**BGN** [ng/mL]	**DCN** [ng/mL]	**AOPP** [ng/mL]	**MMP—12** [μg/mL]
**Healthy children** (HC)	Female (n=14)	102.93* (Q_1–91.99; Q_3 —109.22)	333.36±78.46	53.77* (Q_1—37.58; Q_3—63.54)	5.78* (Q_1—4.75; Q_3—6.29)	19.59±4.67
	Male (n=11)	118.23* (Q_1–88.61; Q_3 —140.42)	389.33±95.62	58.73* (Q_1—49.51; Q_3—71.98)	5.87* (Q_1—5.01; Q_3—9.21)	22.01±4.70
**Children with JIA before treatment** (TB)	Female (n=22)	128.65* (Q_1–107.95; Q_3 184.96)	295.85±38.71	76.32* (Q_1—61.39; Q_3—90.60)	8.77* (Q_1—8.24; Q_3—10.60)	20.39±3.47
	Male (n=12)	130.76* (Q_1—110.11; Q_3 171.92)	292.95±59.88	85.08* (Q_1—64.51; Q_3—123.81)	9.43* (Q_1—8.01; Q_3—10.60)	20.03±3.89
**Children with JIA after 3—6 months of therapy DMARDs** (T0)	Female (n=22)	130.20* (Q_1–86.03; Q_3 —155.14)	346.13±73.65	71.51* (Q_1—47.00; Q_3—87.84)	5.97* (Q_1—4.60; Q_3—7.06)	20.67±5.19
	Male (n=12)	113.36* (Q_1–102.40; Q_3 —140.60)	336.63±66.13	86.78* (Q_1—55.61; Q_3—153.06)	7.90* (Q_1—5.74; Q_3—9.39)	22.99±4.17
**Children with JIA after 24 months of therapy ETA** (T24)	Female (n=22)	148.39* (Q_1–120.13; Q_3 —198.25)	152.94±33.01	48.64* (Q_1—36.41; Q_3—61.90)	6.89* (Q_1—5.82; Q_3—8.29)	20.76±6.44
	Male (n=12)	174.47* (Q_1–125.83; Q_3 —322.65)	154.23±53.65	55.20* (Q_1—44.60; Q_3—61.89)	7.51* (Q_1—6.11; Q_3—8.68)	20.79±7.96

The results are expressed as mean ± SD; * results are presented in the form of median and quartile range (Q1—first quartile; Q3—third quartile);
